# The minimum inhibitory concentration (MIC) and minimum bactericidal concentration (MBC) of silver nanoparticles against *Staphylococcus aureus*

**DOI:** 10.1080/26415275.2020.1796674

**Published:** 2020-07-23

**Authors:** Prashik Parvekar, Jayant Palaskar, Sandeep Metgud, Rahul Maria, Smita Dutta

**Affiliations:** a Pacific Academy of Higher Education, Research University, Udaipur, India; b Department of Prosthodontics, Sinhagad Dental College, Pune, India; c Department of Endodontics, Pacific Dental College, Udaipur, India; d Department of Endodontics, Bhabha College of Dental Sciences, Bhopal, India; e Department of Endodontics, College of Dental Medicine, Qassim University, Kingdom of Saudi Arabia

**Keywords:** *Staphylococcus aureus*, endodontic infections, silver nanoparticles, MIC, MBC

## Abstract

**Aim:**

To determine the minimum inhibitory concentration (MIC) and minimum bactericidal concentration (MBC) of silver nanoparticles against *Staphylococcus aureus (S. aureus)*.

**Methodology:** The antimicrobial efficacy of the silver nanoparticles was determined by the standard methods of Clinical and Laboratory Standards Institute (CLSI). Different concentrations of silver nanoparticles were prepared, and MIC was calculated by tube macro-dilution method. The MBC was determined by the lowest concentration that kills 99.9% of the initial bacterial population. The data were analyzed by ANOVA and Tukey’s *post hoc* test using SPSS software.

**Results:**

The MIC and MBC of silver nanoparticles against *S. aureus* was found to be 0.625 mg/ml.

**Conclusion:**

The result obtained from this study shows that silver nanoparticles have potential bactericidal effects against *S. aureus* at a concentration of 0.625 mg/ml. Silver nanoparticles can be incorporated in the root canal medicaments, sealers and irrigants as it possess potent antimicrobial efficacy against *S. aureus*.

## Introduction

*Staphylococcus aureus* is a facultative anaerobic, immobile, nonsporulated, gram positive coccus, which is a source of periodontal, periapical, and endodontic infections. It is a minor component of oral micro biota but frequently present in the oral cavity and perioral region [1,2]. It is one of the important resistant microorganisms and is frequently associated with failed root canal treatments. Along with *Enterococcus faecalis, Streptococcus mitis, Prevotella* spp. *and Porphyromonas endodontalis,* it plays a role in causing primary and secondary root canal infections [3,4]. It is responsible for a variety of human infections including superficial lesions in the skin and localized abscesses, central nervous system infections, osteomyelitis, invasive endocarditis, septic arthritis, septicemia, pneumonia, and urinary tract infections [5]. Mortality due to bacteremia caused by *S. aureus* is between 13% and 26% [6].

Antibacterial properties of silver have been known since ancient times. Silver vessels were used to preserve water, which was later attributed to the fact that silver ions inhibit bacterial growth by intervening the bacterial DNA functions [7]. Silver in a nanometric scale (less than 100 nm) has different antibacterial properties compared with bulk form of silver metal, like a large effective surface area of individual silver nanoparticles and strong toxicity to a wide range of microorganisms [8].

Previous studies have investigated the antibacterial effect of silver nanoparticles on both gram-positive and gram-negative bacteria such as *S. aureus, Escherichia coli, Pseudomona aeruginosa* and *Staphylococcus epidermid* [9,10]. MIC and MBC of 5 nm silver nanoparticles on *S. aureus* is yet to be established. The aim of the present study was to assess the antimicrobial efficacy of silver nanoparticles against *S. aureus* by determining both MIC and MBC.

## Materials

### Silver nanoparticles formulation

Silver nanoparticles of an average particle size of 5 nm were procured from nanoComposix San Diego, USA, in a solution form. The stock solution was used to prepare six different concentrations of 5, 2.5, 1.25, 0.625, 0.312 and 0.156 mg/ml.

### *S. aureus* strain, medium, and cultivation

The revived ampoule of *S. aureus* (ATCC: 25923) bacteria was centrifuged at 11,000 rpm for 5 min in BHI broth. 20 ml of sterile normal saline was added after discarding the supernatant. The remaining bacterial precipitate was viable and maintained as stock. Then, the concentration was adjusted by spectrophotometer to an optical density of 0.10 at 625 nm (10^8^ CFU/ml, 0.5McFarland’s standard).

## Methodology

### MIC determination

The standard broth dilution method (CLSI M07-A8) was used to study the antimicrobial efficacy of silver nanoparticles by evaluating the visible growth of microorganisms in the agar broth. Serial two-fold dilutions of silver nanoparticles in concentrations ranging from 5 mg/ml to 0.156 mg/ml with adjusted bacterial concentration (10^8^ CFU/ml, 0.5 McFarland’s standard) were used to determine MIC in BHI broth. The control contained only inoculated broth and incubated for 24 h at 37 °C. The MIC endpoint is the lowest concentration of silver nanoparticles where no visible growth is seen in the tubes. The visual turbidity of the tubes was noted, both before and after incubation to confirm the MIC value.

### MBC determination

After the MIC determination of the silver nanoparticles, aliquots of 50 μl from all the tubes which showed no visible bacterial growth were seeded on BHI agar plates and incubated for 24 h at 37 °C. When 99.9% of the bacterial population is killed at the lowest concentration of an antimicrobial agent, it is termed as MBC endpoint. This was done by observing pre and post-incubated agar plates for the presence or absence of bacteria.

### Statistical analysis

The MBC result was analyzed by ANOVA using descriptive statistics including mean and standard deviation. Tukey’s *post hoc* test was done for the analysis of MBC of silver nanoparticles against *S. aureus*. Significance of all the statistical tests was predetermined at *p* < .05.

## Result

### Observations for Staphylococcus aureus

After 24 h of incubation under aerobic conditions at 37 °C, turbidity was noticed in the test tubes 0.156 and 0.312 mg/ml containing silver nanoparticles indicating the growth of bacteria. Whereas in concentrations of 0.625, 1.25, 2.5, 5 mg/ml, no turbidity was seen, exhibiting inhibition of bacterial growth.

The suspension from the tubes of 0.625, 1.25, 2.5, 5 mg/ml was inoculated in BHI agar plate and incubated for 24 h and no growth of bacteria was observed in all the concentrations hence confirming it as bactericidal.

Statistical analysis by ANOVA and Tukey’s *post hoc* test of MBC for different concentrations when assessed for *S. aureus* showed significant inhibition of growth both for 0.625, 1.25, 2.5, 5 mg/ml when compared to 0.156 and 0.312 mg/ml, and the MIC was obtained at 0.625 mg/ml. These results thus confirm that the MIC and MBC of silver nanoparticles for *S. aureus* was found to be effective at dilution of 0.625 mg/ml.

## Discussion

This study was aimed at determining the MIC and MBC of silver nanoparticles against *S. aureus*. The antibacterial effects of drugs are routinely assessed by agar diffusion and MIC test. The advantage of direct contact tests over the agar diffusion method is that it is independent of the diffusion properties of the tested material and media [11]. Serial dilutions of a solution are used for MIC to determine the lowest concentration of material that would still show antibacterial properties.

*Staphylococcus aureus*, a facultative anaerobic gram-positive coccus, has been recovered from several oral sites [12]. It was selected for use in this study because it is one of the facultative bacteria found in failed root canal cases and recurrent apical periodontitis [13] and it can develop resistance to antimicrobial agents.

The antimicrobial effects of silver are mostly attributed to silver ions [14,15]. Silver nanoparticles continuously release silver ions in an aqueous microenvironment [16]. Because of the bigger surface area of silver nanoparticles, they show a stronger and better bactericidal effect [17]. The main reasons for bactericidal properties of silver nanoparticles are interfering with the integrity of the bacterial cell by binding to essential cellular structural [18], particularly to their SH-groups [19,20]. Silver nanoparticles also generate reactive oxygen species (ROS) and free radicals which damage the bacterial cell wall and inhibit the respiratory enzymes [21]. Silver nanoparticles disturb the DNA replication and terminate the bacteria. Silver nanoparticles are biocidal to various gram-positive and gram-negative bacteria [22].

In this study, MIC and MBC of silver nanoparticles against *S. aureus* were determined by macrodilution method and both were found to be effective at 0.625 mg/ml. (Tables 1 and 2) This is the first study in the literature to include the MBC of 5 nm silver nanoparticles against *S. aureus*. One study demonstrated that MIC, MBC of 10 nm silver nanoparticles is in concentrations of 1.35 mg/ml against *S. aureus*. [23] This variation might be due to the methodology used to prepare silver nanoparticles and the size of the silver nanoparticles used. The ultrafine particle size causes its action at lower concentration. In this study commercially available silver nanoparticle was used with the size of 5 nm. Silver nanoparticles with less than 10 nm sizes showed an enhanced antimicrobial effect in a study by Agnihotri et al. They also concluded that compared to other sizes of silver nanoparticles, 5 nm size have the fastest antibacterial activity [24].

**Table 1.** Minimum inhibitory concentration (MIC), turbidity for different concentrations of silver nanoparticles after 24 h.

Dilution of silver nanoparticles	5 mg/m 0.5%	2.5 mg/m 0.25%	1.25 mg/m 0.12%	0.625 mg/m 0.06%	0.312 mg/m 0.03%	0.156 mg/m 0.01%
Set 1	–	–	–	–	+	+
Set 2	–	–	–	–	+	+
Set 3	–	–	–	–	+	+
Set 4	–	–	–	–	+	+
Set 5	–	–	–	–	+	+

Positive (+): Turbidity indicating growth; Negative (–): No turbidity indicating absence of growth.

**Table 2.** Minimum Bactericidal Concentrations (MBC) of silver nanoparticles after 24 h.

Dilution of silver nanoparticles	5 mg/m 0.5%	2.5 mg/m 0.25%	1.25 mg/m 0.12%	0.625 mg/m 0.06%
Set 1	–	–	–	–
Set 2	–	–	–	–
Set 3	–	–	–	–
Set 4	–	–	–	–
Set 5	–	–	–	–

Positive (+): Indicating growth; Negative (–): Indicating absence of growth.

Humberto et al. in their study found that silver nanoparticles are effective broad-spectrum biocides against a variety of drug-resistant bacteria [25]. Various studies have concluded that silver nanoparticles possess antimicrobial effect against many bacteria and fungi, including *S. mutans, C. albicans, P. aeruginosa, E.faecalis,* and *S. aureus*, among others, which could decrease the occurrence of secondary caries, fungical infection, endodontic failure, and dental implant losses [26]. Generally, the tendency to develop bacterial resistance is low towards noble metals [27]. Ellis et al. concluded in their study that *P. aeruginosa* was able to develop resistance to silver nanoparticles while *S. aureus* and *A. baumannii* did not develop resistance to silver nanoparticles [28]. Hosny et al. detected plasmid-mediated silver resistance in clinical bacteria like *P. aeruginosa, A. baumannii* and *S. aureus* isolates [29]. Results from Kaweeteerawat et al. study suggested that silver nanoparticles enhanced the bacterial resistance to antibiotics by promoting stress tolerance through induction of intracellular ROS [30]. It is worth noting, however, that most of the studies are related to exogenous and endogenous silver ion resistance. The release of silver ions by silver nanoparticles is only one of the forms by which silver nanoparticles act as an antimicrobial, as discussed earlier [31]. One side effect of silver is argyria, an irreversible pigmentation of the skin that is mostly an aesthetic concern [32]. Silver nanoparticles also display substantial toxicity against fibroblasts, hepatocytes, osteoblasts or bone-marrow cells [33].

An important limitation of this study is that it was done on planktonic bacteria. The dental infections are mostly polymicrobial, consisting of *Enterococcus, Streptococci, Provotella, Porphyromonas* and many more bacteria. They present different properties in biofilm. Further study is required to assess the antimicrobial effect of silver nanoparticles on dental biofilm.

Most of the time, *in-vitro* results do not correlate with *in-vivo* activity. The results obtained from this study suggest that the effective bactericidal concentration of silver nanoparticles against *S. aureus* is 0.625 mg/ml. The use of silver nanoparticles as an antimicrobial agent against *S. aureus* is possible. Silver nanoparticles can be incorporated into the intra-canal medicaments, root canal sealers and also in irrigating solution to eradicate *S. aureus* from the root canal increasing the success rate of root canal treatments. Further *in vivo* studies are needed to imply this effectively. Future scope of antimicrobial silver nanoparticles in contemporary dentistry includes wide ranges of applications in restorative dentistry, endodontics, implantology, dental prostheses, orthodontics and other dental fields.
